# What are the core features of professional learning community in Chinese preschool teachers’ perspectives: based on grounded theory analysis

**DOI:** 10.3389/fpsyg.2023.1177321

**Published:** 2023-09-12

**Authors:** Jia Yang, Yifan Zhang, Yibin Zhou, Yulin Ji

**Affiliations:** ^1^Department of Early Childhood Education, Fujian Preschool Education College, Fuzhou, China; ^2^College of Education, Fujian Normal University, Fuzhou, China; ^3^School of Humanities and Social Sciences, Fuzhou University, Fuzhou, China

**Keywords:** profession learning community, Chinese background, preschool teachers, core features, grounded theory analysis

## Abstract

**Introduction:**

Professional learning community (PLC) has been concerned as an effective way to promote teacher professional development in China. However, PLC must be optimized due to Chinese culture and education system. This study aimed to explore the features of PLC in preschool teachers’ perspectives and provided theoretical basis for PLC localization practice.

**Methods:**

Twenty-eight preschool teachers were engaged in a PLC, their interview data and personal reflection diaries were collected and analyzed based on grounded theory analysis.

**Results and discussion:**

Five core features of PLC in teachers’ perspectives were extracted in this study, including a common vision, a read-practice-share flow, continuous reflection, distributed leadership, peer and organizational support. PLC’s common vision is to promote teachers’ professional development and children’s development. Teachers learn and reflect in the process of “reading-practice-sharing,” peer support and leadership empowerment play an important role in a sustainable PLC.

## 1. Introduction

In the past two decades, profession learning community (PLC) has been generally discussed and applied as the most effective method (Mesa and Pringle, 2019). Institutions such as the National Commission on Teaching and America’s Future, the National Education Association, the American Federal of Teachers and the National Association of Elementary School Principals have realized its important role in promoting teachers’ profession progress and optimizing teaching quality (Jones et al., 2013). In China, compared with primary and secondary school teachers, preschool teachers have the lowest level of education (China News Network, 2013). Researchers have engaged to apply PLC for improving preschool teacher’s professional ability since 2000. However, the present PLC studies in China are more speculative than empirical (Zhang and Liu, 2018). In addition, due to the influence of culture and national system, the practice of PLC in China has some unique characteristics (Zhan and Wan, 2016). This study focused on the core features of PLC in preschool teachers’ perspectives in China to provide theoretical support for further optimization of PLC practice.

### 1.1. Concept of teachers’ PLC

The concept of PLC came from the learning organization proposed by Senge (1990) in *the fifth discipline*, which contained five disciplines of learning organization including systems thinking, personal mastery, improving mental models, building shared vision and team learning. Based on Senge’s viewpoint, many scholars defined teacher community from different perspective. Hord (1997) considered the teacher community as a school where teachers and leaders sought and shared learning experience and carried out effective learning activities to improve teachers’ professional abilities and students’ learning performance. While others argued that school-based professional communities should include the following three parts: teachers’ reflective dialogue on teaching practice and student learning, observing each other and working together to solve problems and the engagement in practical joint work through partner (Bryk et al., 1999). On this premise, some researchers further refined the concept of PLC: it is a school-wide culture focuses on critical reflection and practice, aims to build a predictable collaboration which is inclusive, authentic and continuous to improve student achievement (Toole and Louis, 2002). Therefore, school-wide activities were oriented toward student performance and based on interpersonal interaction. Teachers’ inquiries and reflections were the main path of PLC (Bolam et al., 2005, 2007; Stoll et al., 2006; Stoll and Louis, 2007; DuFour and Eaker, 2008; Brodie, 2013). In addition, PLC must be driven by a common vision of learning and seek approaches through the community (Stoll et al., 2006). The cohesion of the community, formation of collective knowledge, inner and ethical care between teachers, students and school leaders were all important (Stoll and Louis, 2007).

### 1.2. The features of teachers’ PLC

There have been a wide range of opinions about the features of teachers’ PLC. Bryk et al. (1999) proposed four characteristics of PLC: reflective dialogue, open practice, cooperation and sharing of work, and normative control. Hord (1997) argued that PLC included shared leadership and practice, constant inquiry and learning, and the establishment of cooperative structures or relationships. He later modified the characteristics of PLC as supportive and shared leadership, shared values and vision, collective learning and application, supportive conditions, and shared practices (Hord, 2004). In particular, he highlighted shared values and vision, and set out supportive conditions to facilitate the PLC operation (Hord, 2004). The five features of PLC presented by Hord have been widely mentioned and applied in subsequent studies (Bolam et al., 2005; DuFour and Eaker, 2008; Hipp and Huffman, 2010; Jones et al., 2013; Rincon-Gallardo and Fullan, 2016; Prenger et al., 2017). Many scholars added new features of PLC. For example, Bolam et al. (2005) proposed open network and partnership, inclusive membership, a community culture of mutual trust, respect and support. While others extended the common vision by proposing a shared mission (purpose), vision (clear direction), values (collective commitment), and goals (indicators, timelines, and goals), this visions all focused on student learning and searching for the best practices (DuFour and Eaker, 2008). In addition, researchers focused on the operation of PLC differently. Katz and Earl (2010) proposed the operation of PLC required a challenging focus and effective relationship building through trust, including moderate professional conflict and rigorous research. Some emphasized collaborative teaching and learning, using data to guide decision making and improvement, and actively engaging families and communities in PLC (Jones et al., 2013). Besides, some scholars valued the flat structure of the community and proposed to implement internal accountability and used collaborative investigation cycles to continuously improve PLC practices and systems (Rincon-Gallardo and Fullan, 2016). Others stressed the importance of trust, personal prior knowledge and motivation in PLC implementation (Prenger et al., 2017).

Based on the views of scholars, PLC presented the following core characteristics: (1) Common vision and belief; (2) Establishing student-oriented collective responsibility and partnership; (3) Leadership to support and share; (4) Practice-based cooperation and sharing; (5) Reflective participation and interaction; (6) Material and spiritual support of trust and respect; (7) Network based external links.

### 1.3. The features of teachers’ PLC in China

Chinese scholars have also put forward some characteristics of PLC, including building a common vision, joint leadership and professional autonomy, community cultural construction and teacher professional practice.

Common vision is the emotional basis of shaping PLC. In order to reshape the professional development of teachers, it is necessary to awaken the growth needs, cultivate the awareness of active learning and formulate a common vision of teachers (Niu, 2013; Du and Chang, 2018). Common vision should emphasize deep commitment to students and education, highlighting goal-orienting, co-creation and sharing (He, 2018).

Shared leadership is the leadership mode of PLC. Dialogue, consultation and self-leadership are common ways of leadership (He, 2018). Allowing teachers to share leadership enables a structural shift from administrative control to benefit liberation, promoting collectivism and cooperative learning (Du and Chang, 2018). In addition to sharing leadership, leaders need to improve their professional ability, guidance ability and action ability. They need to respect teachers’ professional autonomy in practice and pay attention to teachers’ efforts in school reform in order to promote teachers’ professional development (Hu, 2013).

The construction of community culture is the ideological foundation for development. Community culture is an ecosystem that includes shared vision, collaborative reflective practices, socially supported tools and resources, learning evaluation of meaning construction (Ru and Cao, 2014). It’s also a culture of “integration, symbiosis and common progress” (Zhang et al., 2016). The community culture of autonomy, equality and cooperation pays attention to the heterogeneity of teachers, which can change the unequal living state of teachers in schools and improve their working enthusiasm. This culture respects the personality of teachers and can deal with contradictions, thus improving teachers’ teaching ability (Hu, 2013; Niu, 2013). Through the profound reflection on their own culture, the construction of teachers’ individual culture can reasonably coordinate the relationship between individual and collective, and promotes the transformation of teachers from individual to group under the active guidance of multi-subject (Liu and Wang, 2016).

Community content should pay attention to the problems existing in teaching practice. The main ways to promote teachers’ professional development include practice reflection, curriculum consciousness change in practice, action exploration in real situation and developmental evaluation in practice. Only by transforming practical knowledge into teachers’ professional knowledge can we promote the development of teachers’ professional competence (Ru and Cao, 2014; Sun, 2019). What’s more, since teacher learning is experiential and participatory, it can promote teachers’ effective learning by crossing community boundaries and highlighting the differences of members (Wang, 2014).

Although the characteristics of PLC proposed by Chinese scholars were similar to those of foreign scholars. Chinese studies ignored the deep respect, collective responsibility, commitment to continuous improvement, and engagement of family and community that Western researchers focused on. The study concerned about the core characteristics of PLC in practical participants’ perspectives, which could further optimize the practice of PLC in China.

## 2. Methods

This study was a single-case holistic design. Interview data and reflective diaries were collected in a PLC, and a PLC assessment scale (PLCA-R) were used to qualify them. The qualitative data were analyzed based on grounded theory, which included data collection, analysis and comparison (Corbin and Strauss, 2008). Open coding, axial and selective coding were used to establish a model of PLC characteristics in preschool teachers’ perspectives.

### 2.1. Participants

Twenty-eight preschool teachers from six inclusive private kindergartens were selected in Fujian, China. They all participated in a-year-long PLC on child language development. Their information of education, teaching experience and job position were presented in Table 1.

**Table 1:** Information of participants.

Code	Age	Teaching age	Duration in service	Position	Education
G102	26	4	4	Grader leaders	College
G103	26	3.5	3.5	Grader leaders	College
C104	27	4.5	4	Coach	College
G105	28	4.5	4.5	Grader leaders	College
C106	25	4.5	4.5	Coach	College
C107	33	12	12	Coach	Bachelor
D108	40	21	15	Director	Bachelor
T109	25	5	4	Teacher	College
T110	23	3	3	Teacher	College
T111	24	6	2	Teacher	Bachelor
T112	26	4	2	Teacher	Bachelor
T201	26	3.5	3.5	Teacher	Bachelor
T202	27	6	1	Teacher	Bachelor
T203	29	6	5	Teacher	Bachelor
T204	26	3	3	Teacher	College
T206	25	2.5	2.5	Teacher	College
G207	36	20	8	Grader leaders	College
T301	34	11	5	Teacher	Bachelor
G302	30	8	7	Grader leaders	Bachelor
T303	29	8	3	Teacher	Bachelor
T304	30	6	1.5	Teacher	Bachelor
T401	24	3	3	Teacher	College
G402	24	5	5	Grader leaders	College
G403	28	4	4	Grader leaders	College
T404	29	3.5	1.5	Teacher	Bachelor
C501	31	12	12	Coach	Bachelor
T502	26	5	1	Teacher	Bachelor
T503	24	1	1	Teacher	College

### 2.2. Interview and reflective diary procedure

Twenty-eight preschool teachers and their principals participated in the interview. They were asked about their PLC experience, emotion, results, progresses and recommendations. Every single interview had exceeded 1.5 h and all voice materials were transformed into scripts and checked by professional transform tools.

An effective reflection should include: implied knowledge in action, knowledge in action and reflection on action (Schon, 1983). In this study, PLC participants kept a diary and reflected on their individual or group activities every week, and 503 reflection diaries were collected. The main reflection include:

Question 1: What did I do today? Who was involved in the incident? Under what circumstances? How did it happen, develop and end? What was the point of this?

Question 2: Why did I think this should be done? What did I expect this to mean? Did my expectations come true? What facts did I find that conflicted with this expectation? Why?

Question 3: What else could or should I have done?

### 2.3. PLC qualification measures

The Professional Learning Community Assessment-Revised scale (PLCA-R) was used to test whether the group complied with the basic characteristics of PLC operation. The scale proposed that teachers’ PLC should include five aspects: shared and supportive leadership, shared value and vision, collective learning and application, shared personal practice and supportive conditions (Hord, 1997). Participants chose “strongly disagree, disagree, agree, strongly agree” based on their perception and identification of the community behavior indicator. The reliability and validity of PLCA-R were tested on 1,209 teachers. Cronbach’s Alpha coefficient of the scale was 0.97 and that of the five aspects was 0.94, 0.92, 0.91, 0.87, 0.82 (relational condition) and 0.88 (structural condition respectively) in sequence (Hipp and Huffman, 2010). The results showed that the average PLCA-R score was 3.76 for six dimensions, and scores of all dimensions were higher than 3.5, which indicated that the case conformed to the basic characteristics of PLC (as shown in Table 2).

**Table 2:** Descriptive analysis of the teacher’s PLC operational quality survey (*N* = 28).

	Mean	STD
Shared and supportive leadership	3.81	0.28
Shared value and vision	3.81	0.31
Collective learning and application	3.86	0.30
Shared personal practice	3.79	0.36
Supportive relational conditions	3.85	0.32
Supportive structural conditions	3.76	0.37
Total (Mean)	3.81	0.30

### 2.4. Qualitative data analysis based on grounded theory

To construct new theories based on grounded theory, this study completed multiple rounds of data analysis through the cyclic process of “data collection, analysis, comparison, re-collection, re-analysis and re-comparison” (Charmaz, 2014). Open coding, axial coding and selective coding were used (Corbin and Strauss, 2008). We used Nvivo 12 Plus to browse the script, and set the free nodes line by line, then created tree nodes for axial and selective encoding, and populated different attributes by category finally. Two researchers completed the coding independently.

#### 2.4.1. Open coding

Open coding conceptualizes data and defines data attributes and direction (Corbin and Strauss, 2008). We read interview materials sentence by sentence and initially coded the relevant interview. It has developed 915 open codes, such as “self-breakthrough, freedom of communication, peer recognition,” which were as close to the participants’ statements as possible (as shown in Table 3). Then we confirmed the name of codes together, coded the same data respectively, and discussed the coding results until reaching a consensus.

**Table 3:** Open coding examples.

Raw data	Open coding I	Open coding II
You can say whatever you want in a group, whatever right or wrong. (G403-D) Because the sharing session is not about criticizing who is right or wrong, nor is it about competing. In the discussion section, we express our opinions on the topic of “the formation of good reading habits and behaviors,” from theories to strategies. (T203-D)	Free communication without judgment	The main manifestations of peer support
At first, I was afraid to express my thoughts. Once, I tried to express my own views on a certain issue. Unexpectedly my companions valued my opinions, and said that my opinions expanded their thinking. It gave me the courage to speak up. (T204-I)	Peers agree with you	
Although opinions differ, everyone will explain to defend his or her own point of view. (T 206-D)	Peers accept different points of view	
Combined with teacher 503’s mind map, I once again found that the language area layout of my class could be adjusted, which I think is very good and practical. (G403-D)	The peer’s speech is enlightening	

#### 2.4.2. Axial coding

In axial coding, all categories are connected with condition, action or interaction, and consequence, which explain “when, where, who, why, how and what” (Corbin and Strauss, 2008). We have reached a common vision for teachers in terms of child development and personal professional development. Twelve categories were developed in this step (as shown in Table 4).

**Table 4:** Axial coding examples.

Axial coding		Open coding
Category 1		The peer support content perceived by teachers and its impact on individuals
Subcategory 1.1	Cause conditions 1.1.1	The convenor inquiries and hopes to induce his companions to speak. The community norm calls for reading and sharing every week;
	Veins conditions 1.1.2	(When) while a partner speaks; When he speaks; When opinion differs among peers; (Where) the PLC activity site; Communication privately. (Who) a companion; The convenor. (How) have never expressed their ideas independently among peers; Not sure what you think is right; Fear of being contradicted;
	Intervention conditions 1.1.3	Convenor to learn inquiry skills, in order to mobilize the enthusiasm of community to speak; Community norm requires active interaction;
Subcategory 1.2	Interaction or action	Free communication without judgment; Peers agree with his/her views; Peers accept different points of view; The peer’s speech is enlightening;
Subcategory 1.3	Consequences	Not afraid to express your mind; Dare to disagree personally; Gain unknown experience from peer’s sharing; Feel relaxed and free; Look forward to the activity;

#### 2.4.3. Selective coding

Selective coding is a process of integrating and refining theories to form theoretical models, and to create a few core categories around which other categories are constructed (Corbin and Strauss, 2008). After the feature model was refined in the discussion, we reviewed the selection of core categories and put forward suggestions for revision. Finally, we have formed a PLC characteristic model of five core categories from Chinese preschool teachers’ perspective.

#### 2.4.4. Validity and reliability

Reliability was tested at different stages of the study. Firstly, the interview data and personal diaries were compared to ensure accurate and consistent. After voice materials of interview were transformed, all scripts were returned to interviewees and confirmed to be authentic and accurate. In order to ensure the objectivity and consistency of the data analysis, two researchers coded data separately. Kappa test was conducted with Nvivo 12 Plus and all coefficients surpassed 0.80, as shown in Table 5.

**Table 5:** Kappa test for consistency.

Code	Document size (Chinese characters)	Kappa
G102	14,599	0.8232
G103	13,186	0.875
C104	10,839	0.9034
G105	22,759	0.9233
C106	20,568	0.8798
C107	18,461	0.8998
D108	15,782	0.9342
T109	10,372	0.9551
T110	13,765	0.8876
T111	14,538	0.8342
T112	14,857	0.9156

## 3. Results

### 3.1. Five core features of PLC

**Figure 1:**
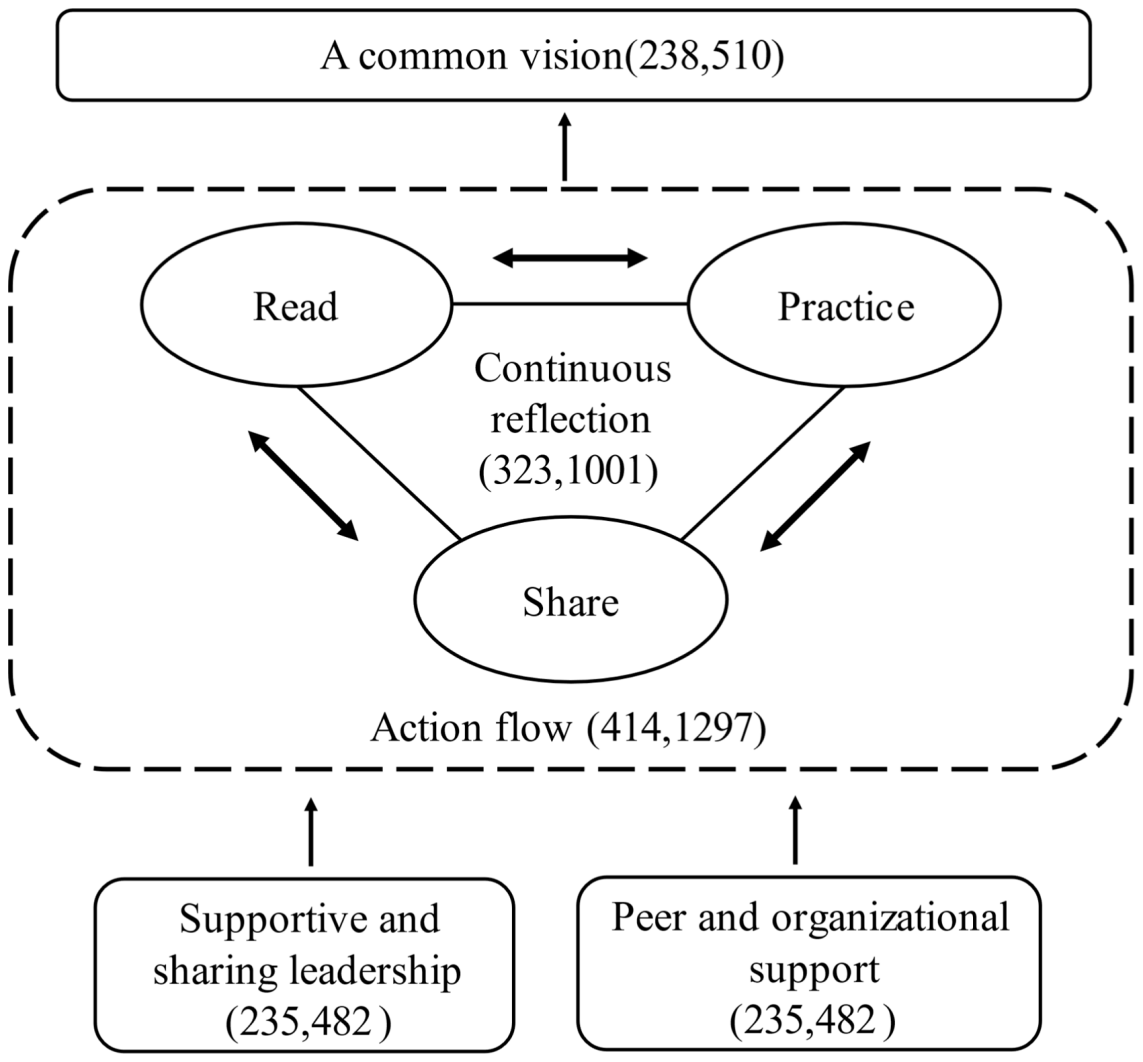
The core features of PLC in the perspective of Chinese preschool teachers. The first number in the parenthesis indicates number of file data involved, while the second indicates number of nodes.

Through open coding, axial coding, and selective coding, 398 open coding formed 12 categories. Five core features of optimal PLC were extracted with Nvivo 12 plus, including a common vision, a read-practice-share flow, continuous reflection, supportive and sharing leadership, peer and organizational support, as shown in Figure 1.

In 476 file data involved, there were 2,146 nodes pointing to peer and organizational support, following by the learning-action stream of “reading-practicing-sharing” (1279), continuous reflection (1001), common vision (510), and finally supportive and sharing leadership (482). Researchers ranked each category by the number of nodes and analyzed each category concretely.

### 3.2. Peer and organizational support for individual learning activities

In axial coding, the researchers clustered the open coding with the “who” in Veins Conditions as the focus, and analyzed the open coding from the aspects of “cause of concern, support mode, support result, emotional experience.” We found that peer support and organizational support played an important role in PLC. Hord (2004) indicated structural and relational supporting conditions in PLC. In this study, participants gave a high response to both supports. In relationship support, peer support provided participants with deeper feelings. In structural support, time guarantee had a great impact on their learning.

#### 3.2.1. Peer support

The most important relationship in PLC is peer relationship, which directly affects the smooth development of community activities. The main peer supports reflected by participants were shown in Table 6.

**Table 6:** The performance content of peer support.

Open coding	Nodes
Peers agreeing with their own views	215
Peers speaking enlightening	199
Agreeing with peers	112
Free communication without judgment	109
Enthusiastic interactions between peers	61
Peers accepting different views	57
Peers solving their own puzzles	26
Peers helping me find problems	26

Peers agreeing with each other is the most important. When teachers shared content, expressed views or showed practical results, the peers expressed recognition through language, expression, action and so on, which would enhance the sharer’s confidence. It also provided sharer with courage to continue learning and sharing. In addition, the sharer returned this affirmation back to others, enabling them to benefit from a community culture of mutual recognition.

“I was afraid to express my thoughts at first. Once I tried to express my opinion on a certain subject. Unexpectedly my companions valued my opinions, and said my opinions expanded their thinking. It gave me the courage to speak up.” (T204-I).

“When I was analyzing children’s picture books, my peers would nod and smile at me, making me feel what I was talking was very valuable, which would make me enjoy analyzing picture books or sharing reading notes with others.” (C104-I).

“When we just started to make the framework of picture book analysis, my partners advised me to try to write something first. At that time, I didn't have many ideas, I had to rely on my experience to sort them out. We added some contents based on my draft. My peers thanked me for drafting and making discussions so productive. And for the first time, I thought I can do it well.” (T109-I).

However, roughly identifying and following goals leads to a lack of community criticism, which will result in making wrong collectively decisions (Westheimer, 1999). According to this study, peers’ acceptance of different views, peers’ help to identify problems, and peers’ enlightening speaks were also important factors. Mutual acceptance among peers was not the same as blind obedience. On the contrary, because of mutual acceptance of common ideas, participants were more willing to express their opinions openly and more accepting of dissent.

“Although opinions differ, each person would explain to defend his or her own point of view.” (T206-D).

“Because the sharing session was not about criticizing who was right or wrong, nor was it about competition. During the discussion section, we would discuss the topic of ‘forming good reading habits and behaviors’ from both theoretical and strategic perspectives. Only in this way could we gain more ideas.” (T203-D).

Agreeing to disagree is not easy. Members must recognize that peer relationships have been established. In order to maintain such relationships, members needed to withhold their opinions and listen to peers.

“As we finally discussed about the reading strategy, I was less insistent on using the scale I had drafted. I wanted to step back and saw what others thought on this scale, rather than sticking to my point of view.” (C501-D).

“I had more control over expression and was more willing to listen to others, which opened my eyes to the diversity of ideas from others.” (C106-D).

#### 3.2.2. Time guarantee

Building a community definitely takes time, place, money, etc. The community in this study was supported by the organization which were also perceived by members. Among them, the biggest feedback from members was the guarantee of time.

“We were very happy to operate a PLC. We also strived to provide necessary support for community development. For example, we offered teachers half a day of community learning each week, and provided transportation and meal subsidies for teachers who studied outside. Preschools with learning spaces would provide refreshments for community teachers and reserve suitable learning spaces. It was fair to say that the principals of several preschools were fully supportive of community learning.” (Chief -I).

Time is the prerequisite for learning activities. There is a lot of practice in community activities. Observing and communicating these practical activities will certainly occupy daily teaching time. Preschool teachers in China are usually required to lead classes all day, and only part of the concentrated teaching time can be used for lesson preparation. The development of community activities would inevitably affect their work.

“I was really worried that I could not juggle work and study. However, assistant principal not only covered the class for me, but also gave me many good suggestions on how to optimize the class work.” (T301-D).

“In the next few weeks, I needed to go to another preschool for four consecutive weeks to see practical activities. Today, I went to communicate with my principal about changing classes, and she soon helped me arrange it.” (T202-D).

In order to protect teachers’ learning time, preschool administrative teachers should share more work. Of course, the members were aware of this and cherished learning opportunities more.

“While teachers were studying, our administrative staffs were covering their class, which actually increased the workload of administrative teachers.” (Chief -I).

#### 3.2.3. Emotional impact of external support on participants

Emotional change is an important factor affecting PLC activities (Zhan and Wan, 2016). Researchers marked the main emotion performance, as shown in Table 7. We found that the top five sources of positive and negative emotions were mostly related to peer and organizational support for individual learning activities.

**Table 7:** First five positive and negative emotion sources.

Category	Open coding	Node number
Positive emotion	Mutual recognition	327
	Learn and gain	209
	Time ensurance for community activities	206
	The peer’s speech is enlightening	199
	Positive interaction	109
Negative emotion	Worrying about not contributing	39
	Worry about no time to study	36
	Sharing is stressful	31
	Peers fail to understand and support ideas	26
	Confused about how to study	24

Among the positive emotions, mutual recognition, peer enlightening speech and positive interaction were associated with peer support. Most members also learned from peers when observing specific subcodes (133).

Among negative emotions, fears of under-contribution, inability to share stress and being misunderstood and supported by peers reflected PLC members’ concerns about peer relationships. Members were eager to get peer approval, and worried that they were not competent for the sharing task or the sharing quality was low, which would lead to peer disapproval of their sharing. These worries could be a motivator for their growth, but could also be a hindrance to their learning.

“I did not read enough. My arguments were mostly based on my own experience and lacked of literature support, which made me feel powerless when I expressed.” (T203-D).

“The reasons why I was afraid of practice sharing were: (1) I did not know what were the best practices in my work, and I did not know how to analyze the good points from ordinary work. (2) My knowledge was not very rich, and I worried that I could not accurately share the bright points of practice with members. I was afraid that my companions did not understand what I said. (3) I felt nervous about speaking in front of so many people.” (T303-D).

In addition, anxiety about not having time to study was a major source of negative emotions. Although the organization had set aside half a day for members, they needed to use their own time to finish reading, practicing or sharing, which also caused negative emotions.

“As much as I wanted to study, I wasn’t sure I could take time off from my family life to do so. Let us just said I would try.” (C501-D).

“These two days coincided with the graduation performance of kindergarten senior class, I have been busy until now. I had to share the reading with the members tomorrow, but I was not ready right now because I really did not have enough time.” (T301-D).

### 3.3. The learning action flow of “read – practice – share”

Focusing on the community activities perceived by the members and following Veins Conditions as a thread, the researchers teased out three main activities, namely “reading, practicing, and sharing,” as shown in Table 8. Through the open coding of the three types of activities, such as “time, place, participants, activity process and activity result,” the main characteristics of the three types of activities, such as “continuous reading, observing peer teaching and sharing personal practice” were found.

**Table 8:** Main learning action perceived.

Category	Open coding	Node number
Read	Read and comprehend shared books together	258
	Write reading notes	45
	Look for information to solve problem	26
	Read material shared by peers	21
Practice	Observe and analyze peer’s teaching activities	475
	Optimize partner’s teaching program	377
	Try to improve personal teaching problems	203
	Use partner’s approach to teaching activities	166
	Carry out teaching activities using book methods	78
	Identify individual teaching problems	58
Share	Share personal practical experience	489
	Share personal problems and solutions	286
	Share personal thoughts on partner’s reading notes	202
	Share personal advice on partner’s problems	199
	Share your reading notes	62
	Share your different opinions	32
	Share your thoughts on the next stage of learning	18

#### 3.3.1. Reading is the main channel to obtain external information

Community reading activities included reading books together in groups, writing reading notes, sharing readings with each other, and finding problem-solving materials. Reading and understanding groups books together was one of the highest perceptions.

“We have found books suitable for our creative reading activities, which provided basis and support for our thinking and learning.” (T201-D).

“My partner reminded me that it was probably because I did not understand the characteristics of children’s reading comprehension that I could not pry developmental needs from children’s responses. I decided to look the language PCK again.” (T202-D).

“The convenor pointed out that there was no point in arguing on the spot and suggested that we each went back and looked for relevant information on page structure or number. I would then try to find relevant information on page structure and number to support my views. I also looked forward to their support for their views.” (C501-D).

When members started a learning topic, encounter difficulties in teaching or disagree with peers, reading would be an important source of help. However, it was not easy to find suitable reading materials. Members obtained books from their own sources, peers or experts, but found that some books did not offer new perspectives, could not solve problems, and were not useful. It may be an urgent problem to select suitable books efficiently.

“I bought the book they recommended, but I did not get the good points in their recommendation. Maybe I have been looking for some intuitive strategies or teaching methods instead of general preaching and concepts.” (T202-D).

“Recently, I have been reading books that needed to be shared. But after reading them, I found that there was little difference in the content of the books, and I could not find anything special worth sharing.” (G106-D).

“I borrowed five books related to reading from the principal, none of which could directly told me what I could use and how to do in teaching.” (T404-D).

#### 3.3.2. Observing peer teaching is the main path to practice

Practice usually includes personal teaching and observing others’ teaching. This study found that observing, analyzing and optimizing peer’s teaching activities were the most important practical activities perceived by community members.

“We carried out the observation activities in the way of One Lesson and Three Studies, and we gained a lot. These activities allowed us to adapt the content and methods of teaching to children’s performance. This adjustment was the crystallization of collective wisdom, allowing everyone to see the feasibility and effectiveness of their own ideas through activities.” (C502-I).

“We entered the scene with an evaluation scale we made before, which enhanced the objectivity of the observation, allowed us to view the activity from the same perspective, and increased the frequency of interaction and communication.” (T201-I).

In the interviews, researchers found that group discussion was an important behavior in the observation activity and a major channel for members to benefit. The observation scale developed by collective discussion provided a necessary theoretical basis for observation and analysis, and the optimized plan of collective discussion provided a carrier for members to continue practice, observation and analysis. As the crystallization of collective wisdom, they made individual practices more supportive and peer interactions more professional.

Although observation activities could be of great benefit to individual professional development, it was difficult to conduct observation activities in school. The main reason was the resistance of the observed teacher. Since the observed teachers were often in the position of being evaluated, the problems exposed will affect the professional recognition teachers. But teachers in this study were able to overcome these effects.

“I was really looking forward to being watched for the first time, because I had tried it in my class and it worked, and it was a group discussion, and I was looking forward to showing it to my peers.” (T301-D).

“After being observed, the focus of everyone’s discussion was on children’s learning performance and the suitability of activity methods. I knew that I still had many problems in activity organization and teacher-child interaction, but everyone did not pay much attention to it. In addition, I also gained a lot of suggestions on optimizing the activity. Such observation gave me the opportunity to practice and demonstrate without discouraging me, so I could do it several times!” (T201-D).

The main reason members were willing to be observed was that the practical content came from the collective discussion, and what was evaluated was the group discussion activity plan, rather than the observers themselves. On one hand, it could reduce the difficulty of designing activities for the observed; on the other hand, it could reduce the pressure of being judged by the observed. In addition, the discovery of personal teaching problems, the use of new teaching methods was also the main practice content of PLC members. In general, the practice of cycling from collective to individual and again from individual to collective was widely used in the community.

#### 3.3.3. Personal practical experience is the main content to share

Sharing has become the most frequent behavior in community activities, and personal practical experience was the most important content shared by members.

“I could use “cannot wait” to express my mood at this time. Today, I tried the method discussed last time in my class. Surprisingly, children found the details in the picture book quickly, and I did not need to remind them hand by hand. I looked forward to sharing my children’s academic performance with my peers.” (T206-D).

“Older teachers could use their experience to help us refine the details. For example, when we were discussing children’s operation cards, a senior teacher reminded us that the cards should be easy for children to operate and should be placed on the back of the operating table in advance, so as to reduce the interference with children’s operation.” (T401-I).

The experience shared by members came from the community practice and personal teaching. Both could increase teachers’ willingness to share and improve the quality of shared content. In addition, the main contents shared by PLC members included their own problems and strategies, reflections on peer reading notes, suggestions for peer problems, different perspectives, and their thoughts on next learning stage.

### 3.4. Continuous reflection

Reflection is limestone of teachers’ professional progress, it confirmed teaching issues, changes teaching strategies and resolves teaching problems (Kwakman, 2003). In the process of focusing on the types of activities, the researchers found that each activity was accompanied by individual or collective reflection. Therefore, the researchers took reflection as the intervention condition of action or interaction to carry out axial coding, and tried to sort out the conditions, contents and results of the participants’ reflection in different activities (as shown in Table 9).

**Table 9:** Coding of reflections of PLC members.

Category	Open coding	Node number
Reflections on reading	Think over someone else’s book report	154
	Combine experience to understand book content	62
	Ask about other’s book reports	34
	Accept others’ opinions on my book report	8
	Reflect on personal reading notes writing	5
Reflections on practice	Reflect on personal practice	174
	Reflect on the practical program	123
	Reflect on the practices of others	67
	Discuss of their practical experiences shared	25
	Respond to other’s discussion about my experiences	9
	Reflect on children’s performance in practice	6
Reflections on oneself	Reflect on personal emotional responses	56
	Reflect on one’s own experience	11
	Reflect on individual learning styles	8
	Reflect on personal reflection methods	4
Reflections on community activities	Reflect on peer participation in activities	173
	Reflect on activity forms	45
	Reflect on how convenor organize activities	26
	Reflect on activity effectiveness	8
	Reflect on activity time	3

Reflections were inseparable from community activities like reading or practicing. Support members’ ongoing learning by reflecting on others’ book reports and group practice was an important motivation. Participants also reflected on the emotional response of individual activities, the peer participation in activities and the activity forms.

To further explore the quality of reflections, researchers used Bloom’s cognitive domain (1956) and its classes to analyze the reflection diaries from six levels of “knowledge, comprehension, application, analysis, synthesis and evaluation.” Knowledge is simple procedural knowledge that has been previously learned by recalling facts, terms, basic concepts, and answers. Comprehension refers to demonstrating an understanding of facts and ideas by organizing, comparing, translating, explaining, describing, and stating the main ideas. Application is the requirement to combine learned knowledge, facts, skills and rules to solve problems in a particular situation or in a different way. Analysis means inferring information by identifying motives or reasons and looking for evidence to generalize. Synthesis is combining information in different ways to form new patterns or propose alternative solutions. Evaluation refers to the evaluation of the value and internal consistency of information, judging its logical flaws, and comparing it to the highest standards. The encoded reflection data are shown in Table 10.

**Table 10:** Analysis of the quality of PLC reflections.

Raw data	Open coding	Node number
With the share, we have a deeper understanding of picture book stories, its origin, background, page design, picture color, educational opportunities, and the fields it can expand, which are all a harvest. (G102-D)	Knowledge	472
My understanding is that picture book itself does not clearly show the time flow. For example, Runaway Town, it just does not show the time, whether its background, characters or details, it does not show any time, it’s timeless. (C104-D)	Comprehension	215
The Chinese word of the whole book is basically ‘hug’. We can use simple plots to bring out the gradual emotional changes, take children to observe different hugging ways of animals, guide children to initiate bud of empathy, and even extend to the discussion about getting lost, etc. (G102-D)	Application	19
The other day, when she said she could provide ‘mirror operation materials’, I did not agree. I would think that materials would affect children’s concentration in regional activities. Mirror operation materials may be used more to stimulate the interest of young children in reading. It seems that 3-4-year-old children will not improve their independent reading with it. (C501-D)	Analysis	284
In my opinion, we should start from the concept, strategy and practical effect of parent–child reading. We can analyze the progressive problems one by one, so as to continuously deepen the topic. (D108-D)	Synthesis	9
The guide manual we have agreed on this week supports our teaching to some extent, but I think we need to set up questions after the activities to ask the children to verify that they are meeting their goals. The setting of questions is often the focus of thinking, and valuable questions are sorted out to a certain extent from the simple to the profound, so as to truly verify the effectiveness of our guide manual. (G102-D)	Evaluation	2

There were relatively more Knowledge, Comprehension and Analysis, and relatively less Application, Synthesis and Evaluation. However, participants could use methods such as comparison, examples, and reasoning in their understanding to improve their ability to critically analyze and come up with innovative ideas. In evaluation, they could discuss how to adjust and improve based on practical results.

### 3.5. Setting up common vision

In *the Fifth Discipline*, Senge (1990) proposed that a common vision was an image or vision shared by people in an organization, which was the core of the integration of various activities. The shared vision was not only the goal of the PLC, but also existed in the behavior of the teachers in the whole operation. Therefore, the researchers used the “target” as the focus of the axial coding, and found that their common vision centralized on children development and personal profession progress.

#### 3.5.1. Centered on children development

It was made clear to all participants that the ultimate goal of the PLC was to promote children’s development. However, teachers of different level expressed it differently. For directors, the goal was specific and conceptualized. When discussing the content of PLC, the directors would focus on children’s performance in activities, and implemented the goal of promoting children’s development.

“PLC was ultimately for the children, … It taught teachers how to teach children, so it was important… We were engaged in education for many years and this was our goal. Children should be our last beneficiary. Whatever we taught, observed, or conducted, we did it for children’s development.” (D108-I).

“Our goal was to discover beauty in child's integrity, language, behavior etc. Maybe a little general, but we set this as a goal. We wanted to make them more beautiful; we expected they have a better future.” (C107-I).

“The kids might exhibit a certain behavior, regular teachers would report back, and we would gather the problem and focus it on one teacher. If she didn't know why the child did it, we would focus on that child.” (C501-I).

Although ordinary teachers identified with and strived to achieve this goal in PLC, they were unable to describe this goal systematically. In general, PLC activities usually stem from teachers’ observations of children. As curriculum and teaching progressed, teachers obtained self-efficiency from centralizing children’s issue and reaction.

The Bench Ladder game created by teacher 204 was not popular. Through teaching practice, she found some problems. She seized the child’s interest and transferred the initiative of the game to the child.

“I asked children to discuss the bench ladder we played in the morning. ‘Do you think it’s funny? Why do you think it’s funny? Why don’t you think it’s funny? And what else do you want to play?’ I tried to give children opportunities for full discussion. The next day, I encouraged them to set up the ladder as they wish. When I gave them enough autonomy, they began to engage in the activity and walking the ladder. And then, more and more children joined in.” (T204-I).

This phenomenon have been mentioned by many teachers, and most of these discussions came from daily observation and reflection of children’s behaviors and reactions. Teachers also used the reflective results to improve their interaction with children. Children were always the focus in this circle of discussion. As T404 said, curriculum and teaching were more meaningful if the subjects were focused on children. Paying attention to children would aware teachers the value of teaching and research, as well as improved their sense of achievement in teaching.

“She expressed the idea that we should observe children, and pay more attention to them. We needed to cast our eyes on children. I tried it when teaching. I observed and recorded how they played, whether they were happy, how they interacted with situations and peers. The Happy Town activity was more meaningful this way.” (G302-I).

#### 3.5.2. Centered on teachers’ profession progress

All members have proposed profession progress as their main goal in PLC. At the beginning of PLC, they expected to effectively solve teaching problems, increase the knowledge reserve in language education and acquire new information language education. At the end of PLC, researchers interviewed them about their gains or changes, the main gains considered by the participants members were shown in Table 11.

**Table 11:** Members’ thoughts on personal gains or changes.

Raw data	Open coding	Node number
Through the sharing of teachers, we have a deeper understanding of more picture book stories, its origin, back story, page design, picture color, educational opportunities, and the fields it can expand, which are all a harvest. (G102-D)	Professional knowledge enriched	66
Now I will carefully check the preparation of activities before organizing activities, which can reduce the garbage time in activities and the interference to children. (G102-D)	Professional competence enhanced	54
I have got a new inspiration and reference since I learned how to write reading notes and the requirements and methods, meanwhile I noticed the most stimulating content of notes in reading and the inspiration to solve practical problems, which can be applied in my future study. (D108-D)	Learning style changed	48
‘To solve the problem and read’, can stimulate learning motivation and drive, set out to actively look for the next stage of books, and find information through a variety of ways and channels. This power is the biggest harvest. (D108-D)	Be willing to learn and read	30
As a convenor, you must get information from members’ speeches, including the number of times they speak, the emotional expression in their speeches, and the quality of their interactions. (T206-D)	Leadership promoted	29
In this community activity, whether it is reading share or practice share are actively entered for, even if I do not understand. Firstly to overcome the fear in your heart. (G103-D)	Get involved and speak up	24
Each group groped in the confusion, with the support of partners, to find a way out… So think about it, my previous anxiety is really unnecessary. After all, learning is not ‘eat into fat by one bite’. (C107-D)	Let go of anxiety and stress	9
Because of my status and position, decision-making language may appear unconsciously. And I feel later, well, it should not be like this. Learning community is more about mutual respect and equality, which requires everyone to speak freely, promote each other’s change and growth, and change our mental model. I cannot express the joy of starting ‘newly’ with everyone. It also inspires confidence and motivation in each other. (D108-D)	Change the behaving way	8
At the beginning, I was really worried that I could not balance the community activities with my work and family life. But after a month, I felt that I could gradually overcome these difficulties as long as I will. (C104-D)	Be able to balance study and work	2

Although the primary concern of teachers after the implementation of PLC was professional progress, the initial expectation of teachers was progress in professional knowledge and competence. However, the improvement in learning style, learning attitude and leadership ability were not previously anticipated. Therefore, as long as teachers have a common vision to promote each other, their personal development will be comprehensive and meaningful.

In the interview, some teachers mentioned the importance of community norms. It was not easy for teachers to take the first step to reading, practicing or sharing. The norm is the common constraint of the members on their learning actions (Senge, 1990), which plays a strong role in urging the learning activities. A common vision requires oversight to realize it. Such norms could indeed affect members’ learning behaviors to some extent.

“The norm required we to read every week. Even if you were not the sharer this week, you should know the book content in advance. This made it necessary for me to set aside time for reading every day, which was a little uncomfortable at first. But that’s why I have something to share in group activities. When I got approval from my peers, all efforts were worthy.” (G302-I).

“We set the rules ourselves, and everyone had to follow them. Fortunately, there were rules that allow me to practice sharing. Otherwise I wouldn't have had the courage to share with so many people.” (T201-I).

### 3.6. Supportive and sharing leadership

In PLC, college leaders share leadership and enhance the leadership of regular teachers, including authority, power and decision making (Hipp and Huffman, 2010). When focusing on coding Cause Conditions, researchers found that many participants mentioned the impact of leadership on their learning. Sowe focused on “leadership,” coding the behavior, results, emotion of the convenor to lead others. All teachers mentioned that they have experienced the transformation of leadership, specifically from institute-guided to teacher-oriented, from result-pursuit to process-focused, and from passive learning to active learning.

“The biggest difference of PLC learning was that we decided what problem to solve, we organized and managed our own learning contents and methods, we practiced and tested our learning quality. When everything was led by ourselves, our learning was no longer passive.” (G105-I).

“I was a convener leader for the first time in my life. Although I was very nervous, I suddenly felt the responsibility of being a leader. I had never listened so carefully to my peers, nor responded so actively to their speeches.” (C104-I).

When power was shared, members could feel the control and responsibility as leaders, which had a positive effect on their learning initiative. However, if their own leadership and management abilities have not been improved, it would not only make themselves feel frustrated, but also make others feel disordered and ineffective learning. In the process of empowerment, only by constantly improving learners’ leadership, expression and learning ability can they truly be competent in leading learners to carry out learning activities.

“The members could leave some time for digestion when they finished their own views and ideas. The convenor did not repeat the views of the speaker, which would confuse others’ understanding, and it was easy to forget what the speaker said.” (C501-D).

“The promotion of our overall goal was not fully considered in previous meetings, and the purpose of this group meeting was not clear. Everyone couldn’t deeply discuss about the focused content, there were more ideas. And the convener couldn’t get the direction and felt a little confused to catch others’ focus.” (G105-D).

“While hosting, I listened to the sharing member, and thought about how to use the propositions and explorations at the same time. So that I cared about one thing but lost another, finally I didn’t know what I was talking about.” (G403-D).

## 4. Discussion

### 4.1. Features of optimal PLC in China

Five core features of optimal PLC were extracted by grounded theory, including common vision, read-practice-share learning flow, continuous reflection, supportive and sharing leadership, peer and organizational support. Former researches generally discussed most of them except transferring of external knowledge. It indicated that the PLC features were similar in different cultures and educational systems.

However, there were some features that were not presenting in this study. For example, Rincon-Gallardo and Fullan (2016) argued that the establishment of internal accountability should be one of the characteristics of PLC. The internal accountability system emphasized the assessment and supervision of PLC quality. Bolam et al. (2005) and Hipp and Huffman, (2010) proposed the core index of PLC quality to monitor PLC quality. In this study, all people were concerned about the process but the effectiveness of PLC. Moreover, when talking about the system support, the leaders did not mention the establishment and implementation of the evaluation system in this study.

### 4.2. Teachers’ professional development and children’s development are the common vision of PLC

Teachers’ PLC should aim at promoting teachers’ professional development, students’ development and improving school management (Cordingley et al., 2003; Stoll and Louis, 2007; DuFour and Eaker, 2008; Hipp and Huffman, 2010).

Community activities should focus on students and learning. Teachers should understand and commit to the common vision and find their own position in realizing the common vision, which contributed to carry out learning activities effectively (DuFour and Eaker, 2008). In this study, teachers all clearly mentioned in diaries or interviews that they hoped to improve their professional knowledge and ability as teachers, and hoped that improvement could affect the development of children. It has been proved that the most profound gain for teachers was the improvement of professional knowledge and ability. At the same time, teachers’ leadership ability and learning attitude were also improved. This coincided with many other scholars, who also put forward that PLC played a positive role in improving teachers’ learning attitude, mastering new learning styles, improving their reflective ability and leadership ability, enhancing their sense of self-efficacy, and promoting their professional knowledge and ability such as learning, teaching and cognitive construction of knowledge (Bryk et al., 1999; Cordingley et al., 2003; Handoyo and Alhasan, 2020; Jia and Yuantao, 2020; Shyam and Helen, 2020).

Student development was fundamental to the shared community vision. “What development do students need? How to evaluate it? What kinds of support do different students need?” Each of the issues should be an important part of a common vision (DuFour and Eaker, 2008). In this study, the PLC members have applied learning results to teaching and promoted child development. But what about the aspects of child development and how to evaluate it? The teachers have not pay attention to it yet and researchers failed to sort out more systematic specific vision for early childhood development.

Only discussion and practice based on children’s learning performance could trigger teachers’ advanced cognitive performance, accelerate the learning process and improve professional competence (Vescio et al., 2008; Prenger et al., 2017). If professional development was not established on the basis of children’s development, then the teachers’ goal of professional development was not clear, the motivation of learning was not strong, and the learning effect could not be verified.

Community norm is a common behavioral expectation formed in a group, which guides individuals acts and behaves in a community (Levine, 2011). It is important to ensure the effective realization of the vision. Effective norm formulation will improve teachers’ constructive role (Levine, 2011; Mesa and Pringle, 2019). This study also found that when the norm specifically and explicitly constrained learning behavior, it could effectively urge behaviors such as learning and sharing. But when the norm was expressed as a promise to act in a certain way, rather than as a belief, member would feel pressures and constraints (DuFour et al., 2019). If it was not treated in time, it would cause negative emotions. Therefore, how to formulate the norm and implement it in a more flexible way was a common issue for PLC members.

### 4.3. Peer support is the foundation of a sustainable community

The operation of PLC required an organizational culture of trust based on collective decision-making, data decision, sharing information and innovation (Hipp and Huffman, 2010). There are organic, contractual, and relational trusts (Bryk and Schneider, 2002). The core of a trust culture should be mutual honesty among peers, seeking assistance, daring to raise and accept questions, willing to assist and give back, appreciating and respecting peers’ ability and experience, etc. (Lencioni, 2022). In this study, the teachers were learning partners who shared the same professional needs and have basic trust. They also shared the same vision and community pact, shared the learning responsibility, and had the contractual trust. They could express their personal ideas freely in the group, discussed their own opinions, shared practices, and had the courage to innovate. They had a strong sense of positive emotional experience among peers and could feel the support and cooperation of peers.

All the descriptions above showed that a better culture of trust has developed within the community. Such culture played a positive role in reducing the conflicts and enhancing the positive emotions among members. It also supported the continuous learning of community members. The findings echoed those of many scholars. Yin et al. (2019) found that trust between teachers had a positive impact on community professional learning. Ding et al. (2019) found that trust played an important role in community learning as a mediator. Bryk and Schneider (2002) found that trust between teachers and with principals would create a safe and comfortable atmosphere for community growth.

There were aspects that community members have yet to feel. Stoll and Louis (2007) argued that a deep respect required all relevant groups in the school (including the principal, teachers, school staff, parents, and community, etc.) to respect each other, and all of them could be deeply involved in the school work as valuable participants and deal with different opinions in an appropriate way. Bolam et al. (2007) argued that PLC supporters should include all teaching staff whom were directly related to teaching, even cleaners, janitors, etc. In addition, parents and the community should join the PLC as supporters and share responsibility for the development of children (Stoll et al., 2006). Since parenting styles may have great impact on children’s development (Lin et al., 2021). In this study, the members were mainly composed of principals and teachers, while others in the kindergarten and children’s parents did not appear in the PLC. During the learning process, these potential roles did not arouse the attention of teachers. This may lose some important opportunities to promote the children development. Particularly, the neglect of parents may affect teachers’ comprehensive cognition of children, also affect the effectiveness of teachers’ teaching.

In order to allowed more relevant groups to have the opportunity to become a PLC member, the use of network technology, expanding the scope of PLC social needed to be considered. With the support of information technology, Bolam et al. (2005) argued that open network communities are also the main characteristics of PLC.

### 4.4. “Read – practice – share” is the main learning way, and reflection is the core

Kwakman (2003) divided teachers’ professional learning activities into four categories. The first is related to reading to collect new knowledge and information or data. The second is called doing or experimenting and used to improve personal professional practice. The third is reflection, which is the cornerstone of professional development. And the fourth category is cooperation about the interaction between teachers. In this study, reading and practice were also the most frequently used learning methods of the members.

Reading is an important way for teachers to obtain external information, but it should not be the only way. External experts, social resource and academic institutions can all become the channels for external knowledge (Lin, 2010). Managers should support PLC members by providing these channels, which will help reduce their searching time and increase the effective use of knowledge (Sena and Shani, 1999).

Practice is an important way to internalize external knowledge. Only when teachers apply that knowledge to solve problems in personal teaching practice can they have the opportunities to reshape their teaching behaviors, improve their teaching concepts, and ultimately affect students’ learning and development (Victoria and Jolyn, 2021). In this study, the teachers had the deepest perception of collective observation, analysis and optimization of teaching activities. They also expressed that such practice had a positive impact on their personal teaching. Collective participatory observation and discussion are of significant help to improve the quality of course evaluation, teaching quality and morale (Gore et al., 2017).

Sharing is the activity that members experience most in peer interaction. Sharing is part of cooperation, which should also include storytelling, mutual assistance, joint work, etc. (Little, 1990). These contents also appeared in this study. For example, some members designed activity plans together, practiced and optimized together, and they felt most impressively with the talks in problem solving. Liu (2019) found that through dialogue teachers created a space that allowed participants to externalize their knowledge and mobilize their implicit knowledge, which would help the flow of tacit knowledge. Similar findings were also found in this study. In this study, teachers optimized their peers’ teaching plans and behaviors by talking, observing, analyzing and sharing their own personal practical experience. Talks externalized teachers’ internalized knowledge and realized mutual learning and development.

Reflection is generally regarded as an important feature to effectively promote the PLC (Kwakman, 2003; Bolam et al., 2005; Stoll et al., 2006; Prenger et al., 2017). In this study, the teachers’ reflection was permeated in various learning activities, such as reading, practice and sharing. They could not only timely reflecte on individual learning actions, but also reflected on community activities and peer behaviors. These reflections were an important cornerstone for members to continue reading, practicing and sharing. However, their reflections focused on emotion rather than events, the reflection was generalized and its cognitive level was mainly knowledge and comprehension. Similar to the conclusions of Liu et al. (2022). They found positive and confused emotions contributed more to higher-level cognition than negative emotions. The reason may be related to the teacher’s specific focus. Vescio et al. (2008) found in their postsecondary research that practice based on students’ learning problems could trigger teachers’ thinking and discussion on deeper teaching phenomena and principles. Damjanovic and Blank (2018) also proposed in their case study that reflecting on students’ learning process would change teachers’ thinking about the nature of teaching. In this study, the content of reflection was more related to their own development. From the perspective of children’s learning, they did not practice and think much. This would affect the depth of teachers’ reflection and the continuity of continuous reflection.

### 4.5. Empowerment is the impetus for teachers’ professional self-improvement

Hord (2004) believed that the core of shared and supportive leadership was to break centralization and support power sharing. In this study, we focused on the characteristics of ordinary teachers, where power of leadership referred to all the teachers when they were responsible and organized activities in the PLC, rather than the influence of teacher positions. All participants all deeply felt the right of leadership, professional autonomy, information sharing, and independent decision-making given by the organization. The granting of these rights obviously promoted the enthusiasm of members to speak and initiative to read, which played a good role in promoting personal leadership, professional ability, sharing and communication ability. This was consistent with the findings of many scholars (Bryk et al., 1999; Bolam et al., 2005; Prenger et al., 2017).

However, the acquisition and use of power by members was influenced by many factors. For example, with less teaching experience, teachers’ speeches were more likely to be influenced by personal professional competence and self-confidence, as well as by the words of authoritative members. When the group trust was insufficient, the voice would be affected by the relationship of community members. When collective decisions were inconsistent with their own decisions, an individual’s right to make autonomous decisions would be affected by the group. Wu (2011) proposed that the implementation of empowerment would be affected by leaders’ subjective consciousness, organizational political environment, organizational trust culture, and organizational scale. Leaders’ support for critical atmosphere, encouragement of reflective practices and acceptance of critical results would affect members’ empowerment and enhancement (Keyes et al., 1999). In this study, members’ competence and confidence, the expert rights with high professional achievements, and even the reference rights of researchers themselves would affect the implementation of empowerment. According to these, the key to empowering was to enhance member personal professional confidence and reduce their unconditional obedience to expert rights and reference rights.

## 5. Suggestion

### 5.1. Adhere teacher’s professional development and children development to the common vision of PLC

Common vision is the direction of community members and the guide of their learning behavior (DuFour and Eaker, 2008). This research confirmed that professional knowledge, leadership, learning attitude and ability were teachers’ common vision and their biggest harvest.

However, the professional development of teachers must be established on the basis of children development. It would help to clarify the common vision of promoting children development by Mastering objectives, specific content and evaluation methods of children development. At the beginning of the PLC, members should first identify the goals and needs of children’s development, and collectively formulated indicators to evaluate children development, and then considered what professional development teachers should obtain based on this goal. These contents made clear that PLC’s common vision could help teachers solve teaching problems and improve personal professional ability.

In addition, community norms drafted by PLC members were external forces that maintained the continuous learning. Community members must formulate a concise and specific community norm, and fulfill the norm with collective commitment to ensure the effective implementation of community activities.

### 5.2. Establish a community culture of trust is the basic guarantee for the sustainable operation of community

The establishment of trust is the result of the joint efforts of PLC members, and it is an important condition for a community to maintain good learning atmosphere. The trust culture needs to provide smooth communication channels. Members have the right to participate and make decisions, and can conduct professional dialogue and share in an environment of mutual respect, trust and care (Jerome, 2011). In this study, teachers were concerned about the community trust, and most of the positive or negative emotions were generated. Therefore, PLC managers should actively build and pay close attention to the community culture, discovering and solving the destructive factors as early as possible, and grasping various opportunities that may promote peer trust. For regular teachers, every participant was the builder, maintainer and beneficiary of trust culture. The formation of trust culture required everyone to be honest with each other and actively seek help, dare to ask and accept questions, be willing to help and feedback, appreciate and respect peers.

In addition, the culture of trust was not limited to the community, and all interest groups in early childhood development should be integrated into such a culture. Especially for parents, only when teachers and parents established a solid mutual trust, could teaching behavior benefit children.

### 5.3. Construct the learning action flow of “read-practice-share” with reflection as the core

The “read-practice-share” action flow could become the main way of community learning, but the way for teachers to obtain external information could be expanded from books to external experts, academic institutions or other social resources. The teachers’ practice could include collective teaching observation and individual teaching practice. In addition to sharing personal experience, teachers could also offer their own teaching wisdom through joint teaching and assisting peers in teaching.

Reflection is the source of motivation for teachers’ continuous learning and the key to interact between implicit and explicit knowledge. In PLC, self-reflection and collective reflection should become important links that teachers attached importance to and actively implemented. Only by improving personal reflection ability and mastering effective reflection methods could we realize the reflection cycle from observation, description, analysis to practice, and finally realize self-cultivation. At the same time, promoting collective public reflection could form a learning wheel of shared meaning, joint planning and coordinated action, so that reflection became the core of collective learning in the PLC.

### 5.4. Strengthen the role of empowerment in PLC

Researchers believed that empowerment could best enhance teachers’ motivation for independent learning from the inside out. First of all, kindergarten leaders were required to be willing to delegate their power and return the leadership, professional autonomy, information sharing, participation, decision-making and learning rights to the teachers, so that every teacher had the opportunity to enjoy these rights. At the same time, the preschools should actively improve the ability of teachers to use these powers. Through training, guiding, instructing and other ways, the competent teachers should have the priority to enjoy all kinds of rights. And through the mentoring system and other ways, more and more teachers had the opportunity to use all kinds of rights, and an organizational culture of sharing power and responsibility would be finally realized.

## 6. Limitations, future directions, and implications

This study has some limitations. First, all participants in this study came from the same PLC, their perspectives were affected by the organizational environment and lack of diversity. So the results of this study should be flexibly applied according to the actual situation of the organization. Future research should attach importance to the learning behavior of community members and adjust the activity content and method timely. In addition, participants tended to focus on successful and impressive cases, some important features may be overlooked. To avoid this phenomenon, more case studies should be carried out to make the perspective of Chinese preschool teachers clearer.

Despite these limitations, this study has theoretical implications. This study concerned the experience of Chinese preschool teachers in PLC activities, identified community learning behaviors that impress teachers, triggered the reconstruction of PLC activities to make it more in line with the actual needs of teachers’ learning. Besides, this research extracted five core features of PLC based on the grounded theory, which provided theoretical guidance for the effective operation of PLC in China. This study also has practical implications. It indicated that teachers’ professional development and early childhood development should be adhered to a common vision. The culture of trust was important for the sustainable operation of community. The learning action flow of “read-practice-share” should be constructed with reflection. And empowerment played an important role in PLC. The findings of this study will provide more guidance for organizers who want to operate PLC. It will also reduce practical problems and improve the effectiveness of PLC.

## Data availability statement

The raw data supporting the conclusions of this article will be made available by the authors, without undue reservation.

## Author contributions

JY co-designed the research, main author of the manuscript, contribution to data collection, main investigator responsible for data analysis, and interpretation. YZha and YJ contribution to data collection and drafted the manuscript. YZho co-designed the research and drafted the manuscript. All authors have read and agreed to the published version of the manuscript.

## Funding

This research was funded by the National Education Sciences Planning Fund of China (Key Topics of the Ministry of Education) project no. DHA220418.

## Conflict of interest

The authors declare that the research was conducted in the absence of any commercial or financial relationships that could be construed as a potential conflict of interest.

## Publisher’s note

All claims expressed in this article are solely those of the authors and do not necessarily represent those of their affiliated organizations, or those of the publisher, the editors and the reviewers. Any product that may be evaluated in this article, or claim that may be made by its manufacturer, is not guaranteed or endorsed by the publisher.
